# Phase II study of epirubicin, oxaliplatin and docetaxel combination in metastatic gastric or gastroesophageal junction adenocarcinoma

**DOI:** 10.1186/1756-9966-28-34

**Published:** 2009-03-09

**Authors:** Luigi Di Lauro, Laura Giacinti, Maria Grazia Arena, Domenico Sergi, Silvia Ileana Fattoruso, Diana Giannarelli, Massimo Lopez

**Affiliations:** 1 Division of Medical Oncology B, Institute for Cancer Research, Rome, Italy; 2 Division of Medical Oncology, San Giuseppe Hospital, Albano Laziale, Italy; 3 Division of Medical Oncology, Toraldo Hospital, Tropea, Italy; 4 Biostatistics Unit, "Regina Elena" Institute for Cancer Research, Rome, Italy

## Abstract

**Background:**

This phase II study was designed to evaluate the activity and safety of a combination of epirubicin, oxaliplatin and docetaxel in metastatic gastric or gastroesophageal junction (GEJ) adenocarcinoma.

**Methods:**

Forty patients with measurable distant metastases received epirubicin 50 mg/m^2^, docetaxel 60 mg/m^2 ^followed by oxaliplatin 100 mg/m^2 ^on day 1 of each 21-day cycle. Primary end point was response rates (RR).

**Results:**

All patients were evaluable. The overall RR was 47.5% (95% confidence interval (CI) 32–63). The disease control was 80%. Median time for response was 6 weeks. Median time to progression was 6.3 months (95% CI 5.4–7.2) and the median overall survival time was 12.1 months (95% CI 10.7–13.5). Grade 3/4 neutropenia occurred in 50% of patients with two episodes of febrile neutropenia (5%). Other non-hematological grade 3 toxicities included sensory neuropathy in two patiens (5%), vomiting and mucositis in two patients (5%) and diarrhea in one patient (2.5%).

**Conclusion:**

The combination of epirubicin, oxaliplatin and docetaxel was found to be effective and well tolerated in patiens with metastatic gastric or GEJ adenocarcinoma and maybe an appropriate regimen to be used in the neoadjuvant setting and with molecularly targeted agents.

## Background

Gastric cancer is still the second leading cause of cancer mortality in the world [1], and it has been estimated that this disease caused in excess of 188,000 deaths in Europe alone in 2006 [2]. Frequently, patients with gastric cancer present with metastatic disease and treatment is essentially palliative. Systemic chemotherapy is able to confer a survival advantage and an improvement in quality of life when compared with supportive care alone [3]. However, median time to progression (TTP) is only 4–5 months, with an overall survival (OS) of 7–9 months [3]. No standard chemotherapy-regimen exists for advanced gastric cancer, but the combinations of cisplatin with fluorouracil (FU) and anthracyclines remain among the most extensively employed regimens, although they are associated with considerable toxicities [4].

Oxaliplatin, a third generation platinum compound, in phase II studies has shown activity in combination with fluoropyrimidines in patients with advanced gastric cancer, with response rates (RR) and median OS ranging from 38% to 65% and 8.6 to 11.4 months, respectively [5-9]. In comparison with cisplatin, oxaliplatin shows a better toxicity profile, which translates to patient convenience. Among taxanes derivatives, docetaxel has emerged as one of the most active agents in gastric cancer, either as single agent or in combination with several other drugs [10]. Recently, we reported a 50% RR and a median OS of 11.2 months in 46 metastatic gastric cancer patients treated with a combination of epirubicin, cisplatin and docetaxel (ECD) [11].

In an attempt to improve on these results, we performed a phase II study substituting, in ECD regimen, cisplatin with oxaliplatin in chemotherapy-naïve patients with metastatic gastric or gastroesophageal junction (GEJ) adenocarcinoma.

## Patients and methods

### Patient Selection

Patients with gastric or GEJ adenocarcinoma with distant metastases not previously treated by systemic chemotherapy were enrolled onto the study. Adjuvant chemotherapy without docetaxel or oxaliplatin was allowed if completed at least 6 months before. Patients were required to have measurable disease, ECOG performance status ≤ 2, life expectancy > 3 months, age between 18 and 75 years, adequate bone marrow (absolute neutrophil count ≥ 1,500/μl, platelet count ≥ 100,000/μl), renal (serum creatinine ≤ 1.5 mg/dl) and liver (serum bilirubin ≤ 1.5 mg/dl) functions, normal cardiac function, absence of second primary tumour other than non-melanoma skin cancer or *in situ *cervical carcinoma, no CNS involvement, no prior radiotherapy in parameter lesions, no concurrent uncontrolled medical illness. The protocol was approved and carried out according to the principles of the Declaration of Helsinki and Good Clinical Practice guidelines, and all patients gave their written informed consent to participate onto the trial.

### Treatment

Treatment consisted of epirubicin 50 mg/m^2 ^by intravenous bolus followed, 15 minutes later by docetaxel 60 mg/m^2 ^diluted in 500 ml of normal saline as 1 h infusion, and oxaliplatin 100 mg/m^2 ^diluted in 500 ml 5% dextrose as a 2 h infusion. All drugs were administered on day 1 of each 21-day cycle. Antiemetic treatment consisted of palonosetron 250 μg plus dexamethasone in a 10 minutes infusion before starting chemotherapy. In addition, orally prednisone premedication was used for prophylaxis of docetaxel-induced hypersensitivity and fluid retention. Granulocyte colony-stimulating factor (G-CSF) was used only as secondary prophylaxis once patients had febrile neutropenia or documented neutropenic infection. Treatment was postponed by a maximum of 2 weeks if the absolute neutrophil count was less than 1,500/μl or the platelet count was less than 100,000/μl. The dose of epirubicin was reduced by 25% of the previous dose in case of grade ≥ 3 stomatitis or diarrhea, whereas oxaliplatin was reduced by 25% in case of grade ≥ 2 peripheral neuropathy or grade ≥ 3 diarrhea, and docetaxel by 25% in case of the following toxicities: grade ≥ 3 neutropenia lasting more than 7 days (or in presence of fever), second incidence of febrile neutropenia despite G-CSF support administered after the first occurrence, grade ≥ 3 diarrhea, and grade ≥ 3 stomatitis.

Chemotherapy was generally administered on an outpatient basis for a maximum of eight cycles for patients with objective responses and of six cycles for patients with stable disease (SD). Treatment was discontinued in case of unacceptable toxicity, treatment delay longer than 2 weeks, disease progression, or patients refusal.

### Pretreatment and Follow-Up Studies

Pretreatment evaluation included clinical history and physical examination, automated blood cell count, biochemical profile, ECG, and computed tomography of thorax and abdomen. Endoscopy was performed only in case of complete remission of all measurable lesions. Blood counts were obtained weekly; biochemical profile was repeated every 3 weeks. All measurable parameters of disease were reevaluated every 6 weeks, and every 2 months during the follow-up period.

### Evaluation of Response and Toxicity

Patients were evaluated for response to chemotherapy every two cycles of treatment. Responses were assessed by at least two observers, and were confirmed by an expert independent radiologist. The RECIST criteria were used to evaluate clinical response [12], and all objective responses were confirmed by CT scans at least 4 weeks after the initial documentation of response. TTP and OS were calculated from the date of first chemotherapy cycle to the date of disease progression, death or last follow-up evaluation, respectively. Toxicity was assessed in each treatment cycle using the National Cancer Institute Common Toxicity Criteria (version 3.0). Peripheral sensitive neuropathy was graded according to an oxaliplatin-specific scale as described previously [13].

### Statistical Methods

The primary end point of this study was to estimate the overall response rate of the regimen. Secondary end points were TTP, OS and safety. The Simon's two-stage phase II design was used to determine the sample size [14]. An interim analysis was carried out when the first 18 assessable patients had been recruited. If more than 4 responses were observed, 15 additional patients were to be recruited; otherwise, the study was to be terminated. If more than 10 responses were observed in the 33 patients, the regimen was considered sufficiently active with a significance level of 5% and power of 80% to be submitted for further evaluation. Seven additional patients were recruited in order to improve the statistical power. TTP and OS were analyzed according to the Kaplan-Meier method, and were updated to 31 December 2008.

## Results

### Patients Characteristics

From June 2006 to February 2008, 40 patients with metastatic gastric or GEJ cancer were enrolled by three oncologic Italian centres. All patients were evaluable for efficacy and toxicity. The pre-treatment characteristics of patients are listed in Table 1. None of the patients had previously received chemotherapy for advanced disease; six patients had received adjuvant chemotherapy without docetaxel or oxaliplatin several months before they entered this study (median, 12 months; range, 8–20 months).

**Table 1:** Patient characteristics

*Characteristic*	*No. of patients*	*%*
Patients evaluable	40	100
Age, years		
Median	65	
Range	34–75	
		
Sex		
Male	24	60
Female	16	40
		
ECOG PS		
0	6	15
1	27	67.5
2	7	17.5
		
Disease location		
Gastric	30	75
GEJ	10	25
		
Histologic type		
Diffuse	19	47.5
Intestinal	15	37.5
Unspecified	6	15
		
Previous adjuvant chemotherapy	6	15
		
Status of primary tumor		
Unresected	28	70
Resected	12	30
		
Predominant site of disease		
Liver	24	60
Peritoneum	8	20
Nodes	4	10
Lung	2	5
Bone	2	5
		
No. of metastatic sites		
1	11	27.5
2	19	47.5
≥ 3	10	25

Abbreviations: ECOG PS, Eastern Cooperative Oncology Group Performance Status;

GEJ, gastroesophageal junction

### Efficacy

**Figure 1:**
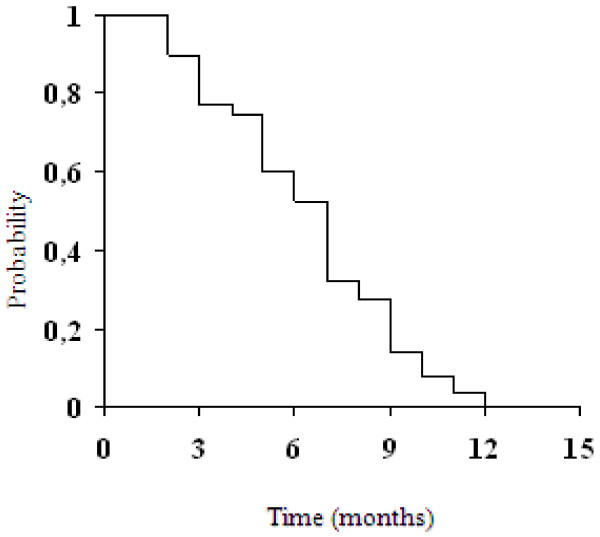
**Time to progression for all patients**.

**Figure 2:**
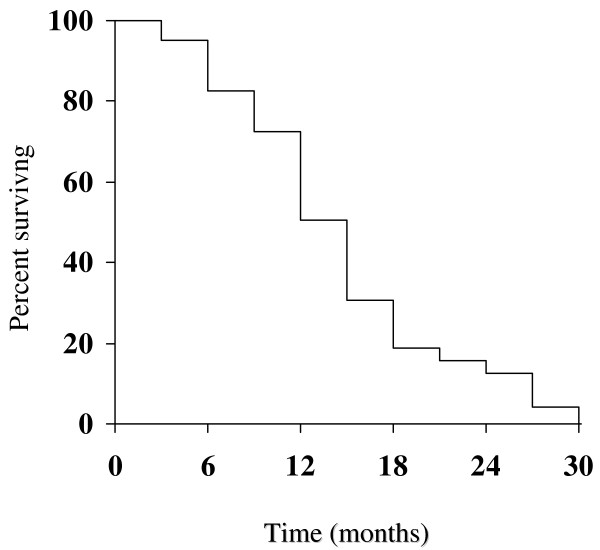
**Overall survival for all patients**.

Among 40 assessable patients, we observed two (5%) complete responses (CRs) and 17 (42.5%) partial responses (PRs), for an overall response rate of 47.5% (95% CI, 32–63). The disease control (CRs plus PRs plus SD) was 80% (Table 2). Responses according to predominant site of disease, were as follows: liver, 12 of 24 patients (50%); nodes/peritoneum 5 of 12 patients (41.7%); lung 1 of 2 patients and bone 1 of 2 patients. Response rates did not significantly differ according to number of metastatic sites: one site, 6 of 11 patients (54.5%); two sites, 9 of 19 patients (47.4%); and three or more sites, 4 of 10 patients (40%). Responses were seen in 2 of 6 patients (33.3%) who received adjuvant chemotherapy and in 17 of 34 patients (50%) not previously treated with chemotherapy. Responses were observed also in 13 of 28 patients (46.4%) with primary tumor not resected and in 6 of 12 patients (50%) with primary tumor resected. RR did non differ when patients were evaluated according to the primary site of disease (gastric: 46.7% and GEJ: 50%, respectively). The median time for response was 6 weeks (range, 6–18). Upon disease progression, 22 patients (55%) received a second-line chemotherapy, including irinotecan/fluorouracil-leucovorin (n = 18) and cisplatin/capecitabine (n = 4). Median TTP was 6.3 months (95% CI 5.4–7.2) (Figure 1). Only 8 patients (20%) progressed within the first two months, whereas at the time of this analysis all but one patient had experienced progressive disease. Median OS was 12.1 months (95% CI 10.7–13.5 months) (Figure 2). One- and 2-year survivals were 50.3% and 12.6%, respectively. Thirty-six patients had died at the time of the present evaluation.

**Table 2:** Objective response in 40 patients

*Response*	*No. of patients*	*%*
Complete response *	2	5
Partial response *	17	42.5
Stable disease *	13	32.5
Progressive disease	8	20

* Disease control: 80%

### Toxicity

Hematological toxicity data are listed in Table 3. A total of 220 cycles of this epirubicin, oxaliplatin and docetaxel (EOD) combination were analyzed in 40 patients, with a median of 6 cycles administered per patient (range, 2–8 cycles). The most important toxicity was myelosuppression, which occurred almost always on day 8 (docetaxel nadir). Grade 3 and 4 neutropenia were recorded in 35% and in 15% of the patients, respectively. Febrile neutropenia occurred in 2 (5%) patients. In these patients a 25% dose-reduction of docetaxel was required, whereas treatment was postponed in 2 (5%) patients and in 7 (3.2%) cycles because of a delay in bone marrow recovery. Mean epirubicin, docetaxel and oxaliplatin dose-intensities were 16.19, 18.48 and 31.90 mg/m^2^/week, respectively, which are equivalent at 97.2%, 92.4% and 95.7% of the planned dose-intensities for these drugs. Grade 3 thrombocytopenia was observed in 2.5% of the patients, and grade 3 anemia occurred in 10% of the patients.

**Table 3:** Grade 3/4 hematological toxicity per cycle and per patient

*Toxicity*	*% of 220 cycles*		*% of 40 patients*	
		
	*Grade 3*	*Grade 4*	*Grade 3*	*Grade 4*
Neutropenia	20	10	35	15
Thrombocytopenia	1	-	2.5	-
Anemia	4	-	10	-

Non-hematological toxicities are listed in Table 4. Mild to moderate transient peripheral neuropathy occurred in 40% of the patients, while grade 3 developed in two (5%) patients. In four of these patients (10%) a 25% dose-reduction of oxaliplatin was required. Alopecia was frequent. Mild nausea and vomiting was encountered in 35% of the patients, and was severe in two (5%) patients. Grade 1/2 diarrhea occurred in 20% of the patients, whereas grade 3 was seen in 1 (2.5%) patient. In this patient a 25% dose-reduction of epirubicin and docetaxel was required. Hypersensitivity reactions, which not precluded chemotherapy continuation, were recorded in 5% of the patients. No cardiotoxicity or treatment-related deaths were observed.

**Table 4:** Non-hematological toxicity in 40 patients

*Toxicity*	*Grade 1%*	*Grade 2* *%*	*Grade 3* *%*
Nausea/Vomiting	20	15	5
Mucositis	10	10	5
Diarrhea	10	10	2.5
Fatigue	20	20	5
Fluid retention*	20	5	-
Alopecia	15	50	35
Neurotoxicity	25	15	5
Hypersensitivity reaction	5	2.5	-

• Grade 1–2: mild; grade 3: severe

## Discussion

This phase II study of triplet cytotoxic therapy for metastatic gastric or GEJ adenocarcinoma showed that the combination of epirubicin, oxaliplatin and docetaxel is an active and well tolerated regimen as first-line treatment. Worth of note are the 47.5% RR, the median TTP of 6.3 months, and above all the median OS of 12.1 months with 50.3% and 12.6% of patients surviving at one year and two years, respectively. In fact, these results were obtained in a very poor prognosis patient population, since liver and/or peritoneal metastases were present in 80% of the cases.

The 1-year survival rate, median survival, and overall rate of response in the present study compare favourably with several chemotherapy regimens including oxaliplatin recently used in advanced gastric cancer. In a four-arm randomized study, 1002 patients with advanced esophagogastric cancer were assigned to receive epirubicin and cisplatin plus either fluorouracil (ECF) or capecitabine, or epirubicin and oxaliplatin plus either fluorouracil or capecitabine (EOX). Although all the treatments were found equivalent, the EOX regimen produced the best outcome with a RR of 47.9% and a median OS of 11.2 months [15]. However, it should be noted that about 25% of the patients had a locally advanced disease as compared to none in our study. In another phase III study, 220 patients were randomized to receive fluorouracil and leucovorin plus either cisplatin or oxaliplatin (FLO). Again, the FLO regimen fared better with a trend toward improved median progression-free survival, but no significant difference in median OS [16]. Apart from peripheral neuropathy, FLO was also associated with significant less toxicity.

A better patient compliance along with an improved tolerability was observed in the present study when compared with our previous similar study in which epirubicin and docetaxel were combined with cisplatin [11]. While RR, TTP and median OS were similar, the inclusion of oxaliplatin reduced toxicity and prevented the need of G-CSF administration.

Although the results of this study are of value in supporting the use of oxaliplatin in gastric cancer, the main question is how the treatment of this disease might be significantly improved in an era in which chemotherapy-related benefits seem to have reached a plateau. Furthermore, current practice is increasingly shifting toward to a more individualized treatment approach. In this regard, several molecularly targeted agents have proved effective in combination with chemotherapy in advanced gastric carcinoma [17].

Given the activity and tolerability, as well as the short time to response (median, 6 weeks), observed in this study, EOD may represent an appropriate regimen to be used also in the neoadjuvant setting and in combination with targeted agents. However, to better define the role of this combination comparative trials with other active regimens in gastric cancer (e.g. EOX, FLO) should be carried out.

## Competing interests

The authors declare that they have no competing interests.

## Authors' contributions

LDL conceived and designed the study, LG, MGA, DS, SIF collected and assembled the data, DG performed the statistical analysis, LDL and ML wrote the manuscript. All authors read and approved the final manuscript.

## References

[B1] Kamangar F, Dores GM, Anderson WF (2006). Patterns of cancer incidence, mortality, and prevalence across five continents: defining priorities to reduce cancer disparities in different geographic regions of the world. J Clin Oncol.

[B2] Ferlay J, Autier P, Boniol M, Heanue M, Colombet M, Boyle P (2007). Estimates of the cancer incidence and mortality in Europe in 2006. Ann Oncol.

[B3] Wagner AD, Grothe W, Haerting J, Kleber G, Grothey A, Fleig WE (2006). Chemotherapy in advanced gastric cancer: a systemic review and meta-analysis based on aggregate data. J Clin Oncol.

[B4] Van Cutsem E, Velde C Van de, Roth A, Lordick F, Köhne CH, Cascinu S, Aapro M (2008). Expert opinion on management of gastric and gastro-oesophageal junction adenocarcinoma on behalf of the European Organisation for Research and Treatment of Cancer (EORTC)-gastrointestinal cancer group. Eur J Cancer.

[B5] Louvet C, André T, Tigaud JM, Gamelin E, Douillard JY, Brunet R, Francois E, Jacob JH, Levoir D, Taamma A, Rougier P, Cvitkovic E, de Gramont A (2002). Phase II study of oxaliplatin, fluorouracil, and folinic acid in locally advanced or metastatic gastric cancer patients. J Clin Oncol.

[B6] Al-Batran SE, Atmaca A, Hegewisch-Becker S, Jaeger D, Hahnfeld S, Rummel MJ, Seipelt G, Rost A, Orth J, Knuth A, Jaeger E (2004). Phase II trial of biweekly infusional fluorouracil, folinic acid, and oxaliplatin in patients with advanced gastric cancer. J Clin Oncol.

[B7] De Vita F, Orditura M, Matano E, Bianco R, Carlomagno C, Infusino S, Damiano V, Simeone E, Diadema MR, Lieto E, Castellano P, Pepe S, De Placido S, Galizia G, Di Martino N, Ciardiello F, Catalano G, Bianco AR (2005). A phase II study of biweekly oxaliplatin plus infusional 5-fluorouracil and folinic acid (FOLFOX-4) as first-line treatment of advanced gastric cancer patients. Br J Cancer.

[B8] Lordick F, Lorenzen S, Stollfuss J, Vehling-Kaiser U, Kullmann F, Hentrich M, Zumschlinge R, Dietzfelbinger H, Thoedtmann J, Hennig M, Seroneit T, Bredenkamp R, Duyster J, Peschel C (2005). Phase II study of weekly oxaliplatin plus infusional fluorouracil and folinic acid (FUFOX regimen) as first-line treatment in metastatic gastric cancer. Br J Cancer.

[B9] Park YH, Kim BS, Ryoo BY, Yang SH (2006). A phase II study of capecitabine plus 3-weekly oxaliplatin as first-line therapy for patients with advanced gastric cancer. Br J Cancer.

[B10] Thuss-Patience PC, Kretzschmar A, Reichardt P (2006). Docetaxel in the treatment of gastric cancer. Future Oncol.

[B11] Di Lauro L, Belli F, Arena MG, Carpano S, Paoletti G, Giannarelli D, Lopez M (2005). Epirubicin, cisplatin and docetaxel combination therapy for metastatic gastric cancer. Ann Oncol.

[B12] Therasse P, Arbuck SG, Eisenhauer E, Wanders J, Kaplan RS, Rubinstein L, Verweij J, Van Glabbeke M, van Oosterom AT, Christian MC, Gwyther SG (2000). New guidelines to evaluate the response to treatment in solid tumours. J Natl Cancer Inst.

[B13] Caussanel JP, Levi F, Brienza S, Misset JL, Itzhaki M, Adam R, Milano G, Hecquet B, Mathè G (1990). Phase I trial of 5-day continuous venous infusion of oxaliplatin at circadian rhythm-modulated rate compared with constant rate. J Natl Cancer Inst.

[B14] Simon R (1989). Optimal two-stage designs for phase II clinical trials. Control Clin Trials.

[B15] Cunningham D, Starling N, Rao S, Iveson T, Nicolson M, Coxon F, Middleton G, Daniel F, Oates J, Norman AR (2008). Capecitabine and oxaliplatin for advanced esophagogastric cancer. N Engl J Med.

[B16] Al-Batran SE, Hartmann JT, Probst S, Schmalenberg H, Hollerbach S, Hofheinz R, Rethwisch V, Seipelt G, Homann N, Wilhelm G, Schuch G, Stoehlmacher J, Derigs HG, Hegewisch-Becker S, Grossmann J, Pauligk C, Atmaca A, Bokemeyer C, Knuth A, Jäger E (2008). Phase III trial in metastatic gastroesophageal adenocarcinoma with fluorouracil, leucovorin plus either oxaliplatin or cisplatin: a study of the Arbeitsgemeinschaft Internistische Onkologie. J Clin Oncol.

[B17] Pozzo C, Barone C (2008). Is there an optimal chemotherapy regimen for the treatment of advanced gastric cancer that will provide a platform for the introduction of new biological agents?. Oncologist.

